# NF-κB inhibitor dehydroxymethylepoxyquinomicin suppresses osteoclastogenesis and expression of NFATc1 in mouse arthritis without affecting expression of RANKL, osteoprotegerin or macrophage colony-stimulating factor

**DOI:** 10.1186/ar2298

**Published:** 2007-09-25

**Authors:** Tetsuo Kubota, Machiko Hoshino, Kazuhiro Aoki, Keiichi Ohya, Yukiko Komano, Toshihiro Nanki, Nobuyuki Miyasaka, Kazuo Umezawa

**Affiliations:** 1 Department of Microbiology and Immunology, Tokyo Medical and Dental University Graduate School of Health Sciences, Tokyo, Japan; 2 Department of Hard Tissue Engineering, Tokyo Medical and Dental University Graduate School, Tokyo, Japan; 3 Department of Medicine and Rheumatology, Tokyo Medical and Dental University Graduate School, Tokyo, Japan; 4 Department of Applied Chemistry, Keio University, Kanagawa, Japan

## Abstract

Inhibition of NF-κB is known to be effective in reducing both inflammation and bone destruction in animal models of arthritis. Our previous study demonstrated that a small cell-permeable NF-κB inhibitor, dehydroxymethylepoxyquinomicin (DHMEQ), suppresses expression of proinflammatory cytokines and ameliorates mouse arthritis. It remained unclear, however, whether DHMEQ directly affects osteoclast precursor cells to suppress their differentiation to mature osteoclasts *in vivo*. The effect of DHMEQ on human osteoclastogenesis also remained elusive. In the present study, we therefore examined the effect of DHMEQ on osteoclastogenesis using a mouse collagen-induced arthritis model, and using culture systems of fibroblast-like synovial cells obtained from patients with rheumatoid arthritis, and of osteoclast precursor cells from peripheral blood of healthy volunteers. DHMEQ significantly suppressed formation of osteoclasts in arthritic joints, and also suppressed expression of NFATc1 along the inner surfaces of bone lacunae and the eroded bone surface, while serum levels of soluble receptor activator of NF-κB ligand (RANKL), osteoprotegerin and macrophage colony-stimulating factor were not affected by the treatment. DHMEQ also did not suppress spontaneous expression of RANKL nor of macrophage colony-stimulating factor in culture of fibroblast-like synovial cells obtained from patients with rheumatoid arthritis. These results suggest that DHMEQ suppresses osteoclastogenesis *in vivo*, through downregulation of NFATc1 expression, without significantly affecting expression of upstream molecules of the RANKL/receptor activator of NF-κB/osteoprotegerin cascade, at least in our experimental condition. Furthermore, in the presence of RANKL and macrophage colony-stimulating factor, differentiation and activation of human osteoclasts were also suppressed by DHMEQ, suggesting the possibility of future application of NF-κB inhibitors to rheumatoid arthritis therapy.

## Introduction

Prevention of bone destruction in affected joints is one of the most important goals in the treatment of rheumatoid arthritis (RA), and many clinical trials of newly developed biologic agents include assessment of radiographic changes before and after treatment. For example, a significant effect of anti-TNF therapy in halting the progression of joint structural damage in active RA has been reported [1-3]. There are still some patients with persistently active disease, however, despite the use of currently available agents; further development of small, cell-permeable agents that specifically interrupt the critical intracellular pathways involved in bone destruction could prove beneficial.

Recent studies have revealed the prominent contribution of osteoclasts to bone resorption that may be dissociated from inflammation in RA pathophysiology. For example, human TNF transgenic mice were protected from bone destruction despite severe arthritis when they were crossed with *c-fos*-deficient mice lacking osteoclasts [4]. In early RA patients treated with methotrexate and infliximab, radiographic progression was slowed even in cases with elevated time-averaged levels of C-reactive protein or erythrocyte sedimentation rate or elevated time-averaged swollen joint counts [3]. Osteoclasts are multinucleated cells formed by fusion of mononuclear progenitors of the monocyte/macrophage lineage. The osteoclasts develop a specialized cytoskeleton that permits them to establish an isolated microenvironment between themselves and the underlying bone, within which matrix degradation occurs by a process involving proton transport to acidify the extracellular microenvironment [5]. Acidification of this compartment leads to the activation of tartrate-resistant acid phosphatase (TRAP) and cathepsin K, which are the enzymes responsible for degradation of bone mineral and collagen matrices [6].

NF-κB is a transcription factor implicated in diverse receptor-mediated signaling pathways including differentiation and activation of osteoclasts [7,8]. Several lines of *in vitro *and *in vivo *studies have demonstrated that inhibition of NF-κB results in suppression of osteoclastogenesis [9-12]. As regards mechanisms underlying the involvement of NF-κB in osteoclastogenesis, Takatsuna and colleagues [12] demonstrated that expression of NFATc1, a key transcriptional factor of osteoclastogenesis induced by macrophage colony-stimulating factor (M-CSF) and receptor activator of NF-κB ligand (RANKL) in a culture of murine precursor cells [13], was inhibited by the NF-κB inhibitor dehydroxymethylepoxyquinomicin (DHMEQ).

DHMEQ is a unique NF-κB inhibitor designed in our laboratory based on the structure of the antibiotic epoxyquinomicin C, which acts at the level of nuclear translocation of NF-κB [14]. An *in vivo *anti-inflammatory effect of DHMEQ has already been demonstrated in various models, including collagen-induced mouse arthritis [15-17]. Since inflammation and bone resorption could be considerably dissociated as mentioned above, and many factors besides RANKL and M-CSF are thought to affect osteoclastogenesis [18], the effect of DHMEQ on *in vivo *osteoclastogenesis needed further investigation. In the present study, therefore, we looked into the effect of DHMEQ focusing on *in vivo *osteoclastogenesis in collagen-induced arthritis. In addition, we tested the effect of this compound on human osteoclast differentiation *in vitro*, to explore the possibility of future development of novel RA therapy.

## Materials and methods

### Inhibitor of NF-κB

The (-)-enantiomer of DHMEQ, which is simply represented as DHMEQ in this manuscript, is a more potent inhibitor of NF-κB than its (+)-enantiomer, and was synthesized as described previously [19].

### Induction of collagen-induced arthritis

Animal experiments were approved by the Institutional Animal Care and Use Committee of Tokyo Medical and Dental University. Male 8-week-old DBA/1J mice were purchased from Oriental Yeast (Tokyo, Japan). Bovine collagen type II (Collagen Research Center, Tokyo, Japan) was dissolved in 50 mM acetic acid at 4 mg/ml and was emulsified in an equal volume of Freund's complete adjuvant (Difco Laboratories, Detroit, MI, USA). Mice were immunized with 100 μl emulsion intradermally at the base of the tail. After 21 days (day 0), the same amount of the antigen emulsified in the same adjuvant was intradermally injected at the base of the tail as a booster immunization. From day 0 to day 10, 100 μg DHMEQ (5 mg/kg body weight) dissolved in 50 μl dimethyl sulfoxide was injected subcutaneously every day to mice in the experimental group. Mice in the control group received 50 μl dimethyl sulfoxide similarly injected.

The thickness of each hind paw was measured on day -4 and on day 10 using a pair of digital slide calipers. Radiographs of both ankle joints were obtained on day 10 and were scored on a scale of 1–3 (1 = no change, 2 = mild osteoporosis without bone erosion, 3 = severe osteoporosis with or without bone erosion) by three investigators who were blinded to the assignment of mouse groups.

### Histochemical staining of osteoclasts

Mice were sacrificed on day 10, 3 hours after the last injection, and their hind paws were excised for experiments. After the skin was scarified with a surgical blade, the left hind paws were preserved in 10% buffered formalin for 3 hours, and the skin was totally removed. The paws were then decalcified in 10% ethylenediamine tetraacetic acid, 5% polyvinylpyrolidone, 100 mM Tris (pH 7.4) for 4 weeks, were dehydrated in graded ethanol, were permeated serially by methyl benzoate and benzene, and were embedded into paraffin in a vacuum oven. Longitudinally sectioned paraffin blocks were fixed in citrate-acetone (2:3 mixture of 380 mM citrate and acetone) for 30 seconds, and were stained with 0.5 mg/ml naphthol AS-BI phosphoric acid and 0.3 mg/ml fast red violet LB salt (Sigma-Aldrich, St Louis, MO, USA) in 27 mM sodium tartrate and 100 mM sodium acetate (pH 5.2) for 1 hour at 37°C. Nuclei were stained with hematoxylin. The mean number of TRAP-positive giant cells with four or more nuclei in the individual ankle joints of arthritic mice was counted under a microscope by two investigators in a manner blinded to the assignment of mouse groups.

### Immunohistological staining of NFATc1 and NF-κB

The right hind paws were frozen in liquid nitrogen, and bone-containing sections were prepared using Cryofilm (Finetec, Tokyo, Japan) [20]. Phycoerythrin-labeled anti-NFATc1 monoclonal antibody 7A6 was purchased from Santa Cruz Biotechnology (Santa Cruz, CA, USA). Monoclonal antibody to the nuclear localization signal in the p65 subunit of NF-κB was purchased from Chemicon (Temecula, CA, USA), was labeled with FITC (Wako, Tokyo, Japan), and was dialyzed against 100 mM Tris, 200 mM NaCl (pH 7.4). After fixing with acetone for 10 min, permiabilization with PBS containing 0.5% Triton X-100 for 10 minutes, and blocking with PBS containing 10% FCS for 1 hour, the sections were incubated with a mixture of the two antibodies for 1 hour, and observed under a confocal microscope (Olympus, Tokyo, Japan).

### Measurement of soluble RANKL, osteoprotegerin and M-CSF

Serum samples were obtained at euthanasia and the concentrations of soluble receptor activator of NF-κB ligand (sRANKL), osteoprotegerin(OPG) and M-CSF were measured by ELISA. The ELISA kits for sRANKL and OPG were purchased from Biomedica (Vienna, Austria), and the ELISA for M-CSF was from R&D Systems (Minneapolis, MN, USA).

### Estimation of RANKL and M-CSF expressed by fibroblast-like synovial cells obtained from patients with RA

Synovial tissues were obtained at the time of total knee joint replacement from six patients with RA; these patients were female, aged (mean ± standard deviation) 67.3 ± 9.1 years, and their serum C-reactive protein levels were 3.4 ± 2.4 mg/dl. Of the six RA patients, five women took prednisolone, four women took methotrexate, two women took bucillamine, and one woman took leflunomide. Signed consent forms were obtained prior to the operation, and the experimental protocol was approved in advance by the Ethics Committee of Tokyo Medical and Dental University. RA was diagnosed according to the criteria of the American College of Rheumatology [21].

RA fibroblast-like synovial cells (FLS) were prepared from the synovial tissues as described previously [22], and were cultured in DMEM with heat-inactivated 10% FCS (Sigma-Aldrich). After incubation with or without DHMEQ for 24 h, RA-FLS were collected and lysed with RIPA lysis buffer (Upstate, Lake Placid, NY, USA). After debris was eliminated by centrifugation, 5 μg proteins in the supernatant were separated by 10% SDS-PAGE under reducing conditions, and were transferred to a polyvinylidene difluoride membrane. The membranes were blocked with 4% Block Ace (Snow Brand Milk Products, Sapporo, Japan) in PBS containing 0.1% Tween 20 overnight, were incubated with anti-RANKL monoclonal antibody 70513 (R&D Systems) or with anti-β-actin monoclonal antibody AC-15 (Sigma-Aldrich) in PBS containing 0.1% Tween 20 with 0.4% Block Ace for 1 hour, and were then incubated with peroxidase-conjugated rabbit anti-mouse IgG (Dako Cytomation, Carpinteria, CA, USA) for 1 hour. The immunoblots were detected by enhanced chemiluminescence (Amersham Pharmacia Biotech, Piscataway, NJ, USA). To analyze M-CSF production by RA-FLS, cells (2 × 10^4^/well) were cultured in 96-well plates with or without DHMEQ for 24 hours. The culture supernatants were collected and the concentration of M-CSF was measured using an ELISA kit (BioSource, Camarillo, CA, USA).

### Estimation of human osteoclastogenesis and production of matrix metalloprotease-9

Peripheral blood mononuclear cells from healthy donors were collected by Ficoll-Conray gradient centrifugation, and monocytes were positively selected using MACS microbeads (Miltenyi Biotec, Auburn, CA, USA). The monocytes (5 × 10^4^/well) were incubated in 96-well plates in αMEM with heat-inactivated 10% FCS (Sigma-Aldrich), 25 ng/ml M-CSF (Peprotech, Rocky Hill, NJ, USA) and 40 ng/ml RANKL (Peprotech). The indicated concentration of DHMEQ was added throughout the culture period. On day 3 the medium was replaced with fresh medium. After incubation for a further 4 days, the number of TRAP-positive multinucleated cells with three or more nuclei was counted under a microscope. To analyze the expression of matrix metalloprotease-9 (MMP-9), osteoclasts were differentiated as above without DHMEQ; then DHMEQ was added after medium replacement at day 7, and the culture supernatant was collected at day 8. The concentration of MMP-9 in the supernatant was measured using an ELISA kit (GE Healthcare Bio-Sciences, Tokyo, Japan).

## Results

### Suppression of *in vivo *osteoclastogenesis by DHMEQ

**Figure 1:**
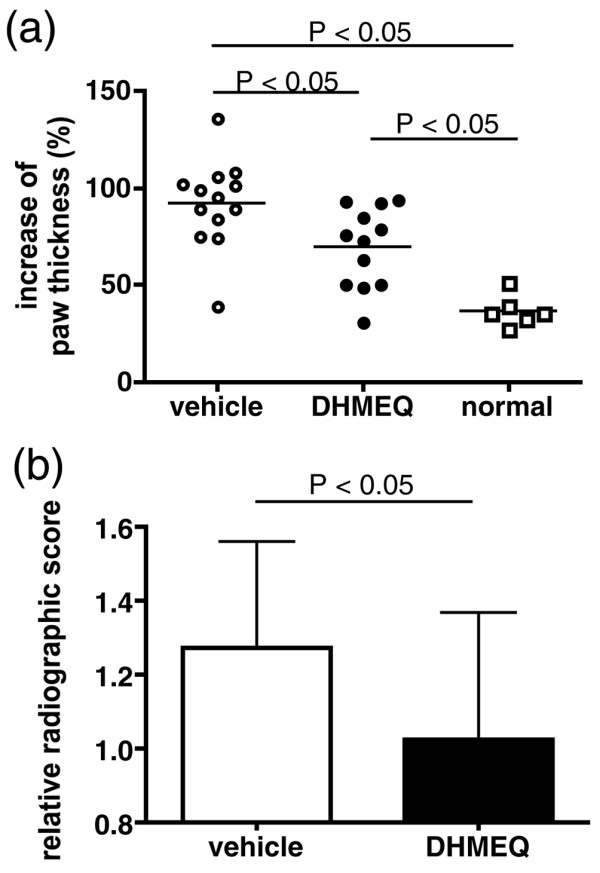
Effect of dehydroxymethylepoxyquinomicin on inflammation and bone destruction in collagen-induced mouse arthritis. **(a) **Increase (%) of the sum of the thickness of the right and left hind paws in each mouse during day -4 and day 10. Horizontal bars represent the mean. DHMEQ, dehydroxymethylepoxyquinomicin. **(b) **Radiographic scores of the ankle joints were determined as described in Materials and methods, and were normalized to the normal mice. Values are expressed as the mean ± standard deviation, and represent data obtained by three independent investigators. Data were compared by Student's *t *test.

We first confirmed the effect of DHMEQ on collagen-induced arthritis by comparing the paw thickness and radiographic changes in the mice treated with DHMEQ (*n *= 12) and vehicle alone (*n *= 13), as well as in nonimmunized age-matched normal mice (*n *= 6), 10 days after booster immunization. As shown in Figure 1, treatment with DHMEQ ameliorated both inflammation and bone destruction.

**Figure 2:**
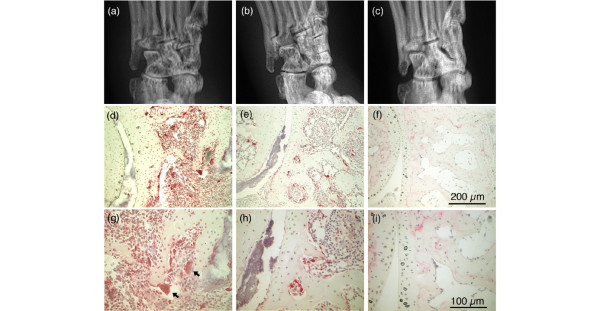
Effect of dehydroxymethylepoxyquinomicin on differentiation of osteoclasts in ankle joints of mice with collagen-induced arthritis. After taking radiographs (a-c), the ankle joints were histochemically examined for tartrate-resistant acid phosphatase-positive cells (d-i). **(a)**, **(d) **and **(g) **Typical joint of an arthritic mouse treated with vehicle alone. **(b)**, **(e) **and **(h) **Typical joint of an arthritic mouse treated with dehydroxymethylepoxyquinomicin. **(c)**, **(f) **and **(i) **Joint of an age-matched normal mouse. Arrow, multinucleated giant osteoclasts.

To examine the effect of DHMEQ on *in vivo *differentiation of osteoclasts, the ankle joints of the mice were excised and processed for histochemical staining. The specimens from arthritic mice treated with vehicle alone showed marked synovitis accompanying invasion of pannus into the marrow space (Figure 2d). Numerous TRAP-positive cells were attached on the eroded bone surface and the inner surfaces of bone lacunae, and some of them were multinucleated (Figure 2g). Radiographically, the ankles of these mice showed remarkable periarticular osteoporosis and bone erosion (Figure 2a). In contrast, the joints of arthritic mice treated with DHMEQ showed milder synovial inflammation. Osteoclasts were mainly observed on the inner surfaces of the bone marrow, and their number and size were less than those in vehicle-treated mice (Figure 2e, h). Radiographs showed mild periarticular osteoporosis (Figure 2b). In ankle joints of normal control mice, virtually no TRAP-positive cells were observed (Figure 2f, i).

**Figure 3:**
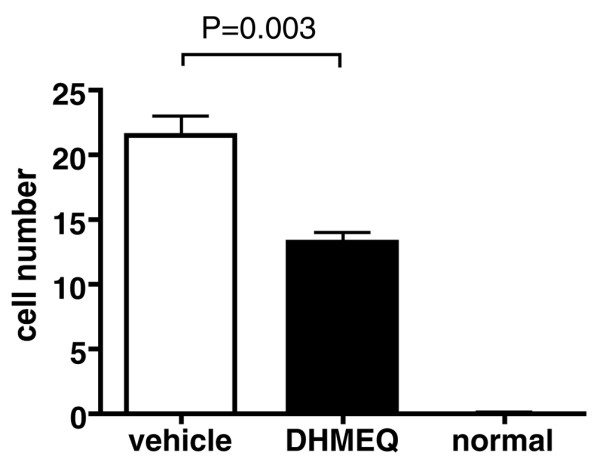
Quantitative estimation of the suppressive effect of dehydroxymethylepoxyquinomicin on *in vivo *osteoclastogenesis. The mean number of tartrate-resistant acid phosphatase-positive giant cells with four or more nuclei in the individual ankle joints of arthritic mice treated with vehicle alone (*n *= 13), of mice treated with dehydroxymethylepoxyquinomicin (DHMEQ) (*n *= 12), and of normal mice (*n *= 6) were counted under a microscope by two investigators in a blinded manner to the assignment of mouse groups. The results shown are the mean ± standard error of the mean of four independent counts, and were compared by Student's *t *test.

For quantitative evaluation, the number of TRAP-positive giant cells with four or more nuclei in each ankle joint was counted. As shown in Figure 3, the mice treated with DHMEQ exhibited significantly fewer osteoclasts than those given vehicle alone, indicating the suppressive effect of DHMEQ on *in vivo *osteoclastogenesis.

### Effect of DHMEQ on production of sRANKL, osteoprotegerin and M-CSF

**Figure 4:**
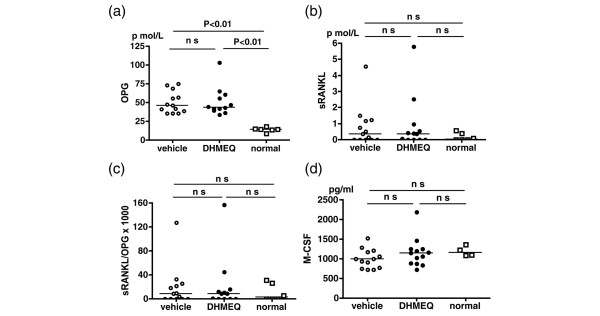
Effect of dehydroxymethylepoxyquinomicin on serum factors involved in osteoclastogenesis. Effect of dehydroxymethylepoxyquinomicin (DHMEQ) on serum levels of **(a) **osteoprotegerin (OPG), **(b) **soluble receptor activator of NF-κB ligand (sRANKL), **(c) **sRANKL/OPG ratio and **(d) **macrophage colony-stimulating factor. Serum levels of these cytokines in individual arthritic mice 3 hours after the last treatment with vehicle alone (*n *= 13) or with DHMEQ (*n *= 12), and in age-matched normal mice (*n *= 4–6), were determined by ELISA. Horizontal lines represent the median. Data were analyzed by the Mann-Whitney test. *P *< 0.05 was considered significant; ns, not significant.

Many *in vitro *studies adopt a culture system in which monocyte/macrophage precursor cells are stimulated with sRANKL and M-CSF for induction of osteoclasts. In the *in vivo *bone metabolism, the naturally occurring decoy receptor OPG also plays a key role by preventing the binding of RANKL to its receptor, receptor activator of NF-κB (RANK). The ratio of circulating OPG to sRANKL in early RA patients has been demonstrated to predict later joint destruction [23]. We therefore tested whether administration of DHMEQ changed expression of these soluble factors. As shown in Figure 4a, serum levels of OPG in arthritic mice treated with DHMEQ and with vehicle alone were both significantly higher than those of normal mice. sRANKL in both arthritic groups also tended to be higher than nonarthritic normal mice, although this was not statistically significant (Figure 4b). No differences in OPG, sRANKL or the sRANKL/OPG ratio were observed, however, between the DHMEQ-treated and vehicle-treated groups (Figure 4a–c). In addition, no significant difference was observed in the serum levels of M-CSF among three groups (Figure 4d).

**Figure 5:**
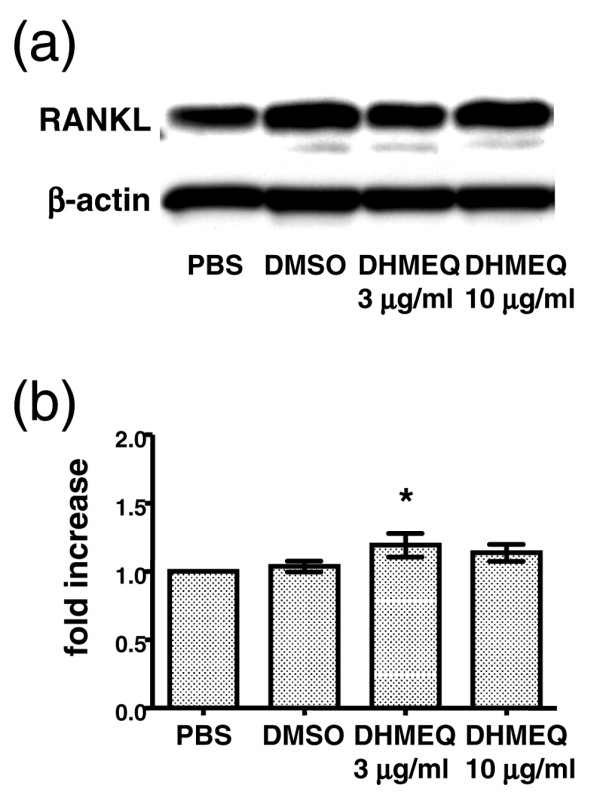
Effect of dehydroxymethylepoxyquinomicin on human fibroblast-like synovial cells. Effect of dehydroxymethylepoxyquinomicin (DHMEQ) on expression of receptor activator of NF-κB ligand (RANKL) and of macrophage colony-stimulating factor (M-CSF) by fibroblast-like synovial cells obtained from patients with rheumatoid arthritis (RA-FLS). The RA-FLS were incubated with DHMEQ, with vehicle (dimethyl sulfoxide (DMSO)), or with PBS for 24 hours. **(a) **Cell lysates were analyzed by western blotting with anti-RANKL or with anti-β-actin monoclonal antibody. Representative data of similar results obtained using cell lines from two patients with RA are shown. **(b) **Concentration of M-CSF in the culture supernatant measured by ELISA; results expressed as relative values compared with PBS. Data are the mean ± standard error of the mean of independent experiments carried out in triplicate using cell lines obtained from six patients with RA, and were compared by Student's *t *test. *P < 0.05.

To further examine the effect of DHMEQ on expression of RANKL and M-CSF, we carried out *in vitro *experiments using human RA-FLS. As shown in the results of western blotting, RA-FLS spontaneously expressed RANKL without the addition of proinflammatory cytokines, and incubation with DHMEQ did not change the level of RANKL expression (Figure 5a). Similarly, the result of ELISA revealed that RA-FLS secreted M-CSF without any stimulation. Incubation with DHMEQ did not suppress the levels of M-CSF, but rather enhanced it slightly at 3 μg/ml (Figure 5b). Stimulation with TNFα did not further increase the production of RANKL or M-CSF by RA-FLS (data not shown). These results suggest that production of RANKL and M-CSF by proliferating RA-FLS are not particularly dependent on NF-κB, and the suppressive effect of DHMEQ on osteoclastogenesis resulted from the downregulation of proosteoclastogenic factors other than RANKL, RANK or OPG.

### Suppression of NFATc1 expression by DHMEQ in arthritic joints

**Figure 6:**
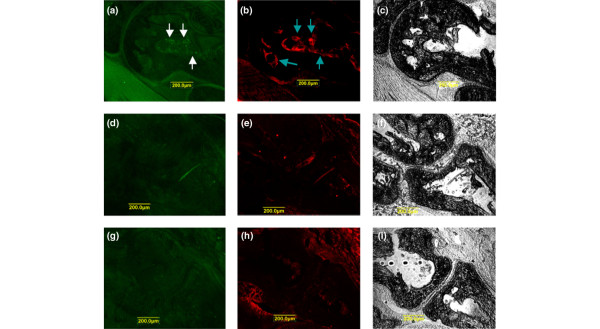
Effect of dehydroxymethylepoxyquinomicin on NF-κB activation and NFATc1 expression in joints of collagen-induced arthritis. Fresh frozen sections of each ankle joint were double-stained: **(a, d, g) **with FITC-labeled antibody to an activated form of the p65 subunit of NF-κB, and **(b, e, h) **with phycoerythrin-labeled antibody to NFATc1. **(c, f, i) **Transmission microscopy images of the same slides to show the articular structure. First row, a typical joint of an arthritic mouse treated with vehicle alone (a-c). Second row, a typical joint of an arthritic mouse treated with dehydroxymethylepoxyquinomicin (d-f). Third row, a joint of an age-matched normal mouse (g-i). White arrow (a), staining by anti-NF-κB p65 antibody; blue arrow (b), staining by anti-NFATc1 antibody of the cells along the inner surfaces of bone lacunae.

In the presence of RANKL and M-CSF, DHMEQ inhibits differentiation of osteoclasts in cultures of mouse bone-marrow-derived monocyte/macrophage precursor cells by downregulation of NFATc1 [12]. We therefore examined the expression of NFATc1 as well as NF-κB in the joints of arthritic mice by immunofluorescent staining. Using monoclonal antibody that recognizes only an activated form of the p65 subunit of NF-κB, distinct staining was observed along the inner surface of bone lacunae (Figure 6a) and in eroded regions of arthritic bone from mice treated with vehicle alone, but was not observed in those mice treated with DHMEQ (Figure 6d). Staining of NFATc1 was also obvious on the inner surfaces of bone lacunae (Figure 6b) and in the eroded regions of vehicle-treated mice, but not from DHMEQ-treated mice (Figure 6e). Normal control mice exhibited no staining of NF-κB (Figure 6g) nor of NFATc1 (Figure 6h). These results suggest that inhibition of NF-κB activation by DHMEQ leads to suppression of NF-κB-dependent expression of NFATc1 by osteoclasts in arthritic joints.

### Suppression of human osteoclastogenesis and MMP-9 expression by DHMEQ

**Figure 7:**
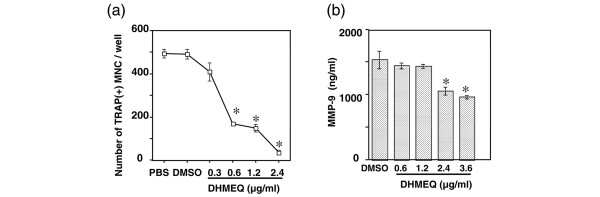
Effect of dehydroxymethylepoxyquinomicin on human osteoclastogenesis and production of matrix metalloprotease-9 by human osteoclasts. **(a) **Peripheral blood monocytes were incubated in 96-well plates with macrophage colony-stimulating factor (M-CSF), receptor activator of NF-κB ligand (RANKL), and the indicated concentrations of dehydroxymethylepoxyquinomicin (DHMEQ). At day 7, the total number of tartrate-resistant acid phosphatase (TRAP)-positive multinucleated cells (MNC) with three or more nuclei/well was counted. Representative data of three independent experiments are shown. *P < 0.01, DHMEQ versus dimethyl sulfoxide (DMSO). **(b) **Peripheral blood monocytes were incubated in 96-well plates with M-CSF and RANKL without DHMEQ. At day 7, the medium was replaced with fresh medium and the indicated concentrations of DHMEQ were added. The culture supernatant was collected at day 8, and the matrix metalloprotease-9 (MMP-9) concentration was measured by ELISA. Representative data of two independent experiments are shown. Data represent the mean ± standard error of the mean of triplicate wells, and were compared by Student's *t *test. *P < 0.05, DHMEQ versus DMSO.

Human peripheral blood monocytes cultured with M-CSF and RANKL differentiate into osteoclasts [24]. To test whether the suppressive effect of DHMEQ on osteoclastogenesis can be applied to human cells, monocytes from peripheral blood of healthy volunteers were cultured with DHMEQ together with M-CSF and RANKL. The result showed that the number of TRAP-positive multinucleated cells was decreased by incubation with DHMEQ in a dose-dependent manner (Figure 7a).

MMP-9 is one of the enzymes released by osteoclasts, and the enzyme plays a role in degradation of the extracellular matrix. Its expression is reported to be modulated by NFATc1 [25], and to be upregulated in serum of patients with active RA [26]. To examine the effect of DHMEQ on MMP-9 production by human osteoclasts, DHMEQ was added to the culture after formation of mature osteoclasts and secreted MMP-9 was measured. The results showed that concentration of MMP-9 in the culture supernatant was partially but significantly decreased by DHMEQ (Figure 7b). These results indicate that DHMEQ suppresses osteoclast differentiation from human peripheral blood monocytes as well as the activity of mature osteoclasts.

## Discussion

In the present study, we investigated the effect of DHMEQ on *in vivo *osteoclastogenesis using a mouse arthritis model, and showed that DHMEQ significantly suppresses differentiation of osteoclasts in arthritic joints. Serum levels of sRANKL, OPG and M-CSF, and the sRANKL/OPG ratio, were not affected by this treatment regimen with DHMEQ, whereas expression of NFATc1 in the joints was suppressed in DHMEQ-treated mice. In accordance with these observations, spontaneous expression of RANKL and M-CSF in cultures of RA-FLS were not suppressed by DHMEQ in concentrations at which it has been demonstrated to suppress expression of proinflammatory cytokines [16]. These results indicate that in a RANKL/RANK/OPG signaling cascade, expression of NFATc1, a key downstream regulator of this cascade, is more susceptible than that of upstream molecules to treatment with DHMEQ.

Expression of RANKL and OPG is coordinated to regulate bone resorption positively and negatively by controlling the activation state of RANK on osteoclasts. The crucial role of the RANKL/RANK/OPG signaling pathways in regulating bone metabolism is underscored by findings that genetic mutations that activate RANK and that inhibit the RANKL binding properties of OPG are associated with familial expansile osteolysis [27] and with juvenile Paget's disease [28], respectively. In addition, a cyclic peptide with sequence homology to a predicted ligand contact surface on RANK has been reported to inhibit RANKL-induced signaling and osteoclastogenesis [29]. Proinflammatory cytokines, such as TNFα and IL-1, are thought to modulate this system primarily by stimulating M-CSF production (thereby increasing the pool of preosteoclastic cells) and by directly increasing RANKL expression [30]. Aberrant expression of RANKL especially on RA-FLS stimulated by TNFα or IL-1 is therefore supposed to be the main contributor to bone destruction in active RA [31].

We previously demonstrated that 10 μg/ml DHMEQ suppresses expression of IL-1β and IL-6, as well as CC chemokines CCL2 and CCL5, in culture of TNFα-stimulated RA-FLS [16]. We therefore speculated that expression of M-CSF, RANKL, OPG, or the sRANKL/OPG ratio may be modulated in DHMEQ-treated mice. Serum levels of these factors, however, were not significantly affected by treatment with DHMEQ. Even *in vitro*, the expression of RANKL and M-CSF by RA-FLS was not enhanced by TNFα, and was not suppressed by 10 μg/ml DHMEQ. Taken together, FLS of RA patients – and presumably of mice – are suggested, once activated, to express RANKL and M-CSF rather constitutively, and they are resistant to treatment with DHMEQ. The ineffectiveness of DHMEQ on RANKL suppression may possibly be ascribed to insensitivity of the transcription mechanism of the RANKL gene to DHMEQ. Regulation of the rate of gene expression is a complex process involving several transcription factors and gene activator/repressor proteins. For example, it has been recently reported that NF-κB collaborates with other transcription factors (early growth response-2 and early growth response-3) in expression of the RANKL gene [32]. Even in molecules whose expression is demonstrated to be NF-κB dependent in a certain assay condition, therefore, the molecules' dependency on NF-κB or sensitivity to DHMEQ treatment varies among the molecules under other conditions. The second possible reason may involve the stability of RANKL once expressed on the surface of FLS. We detected RANKL by western blotting in the lysates of RA-FLS that had been cultured for a few weeks without addition of proinflammatory cytokines (Figure 5); this is consistent with the observation of other investigators [33].

Downstream of the RANKL/RANK/OPG system, a significant part of the genetic regulation of osteoclastogenesis is performed by NF-κB. The critical role of this transcription factor is underscored by the report of Franzoso and colleagues that mice lacking the p50 and p52 subunits of NF-κB develop osteopetrosis [7]. A few years later, the same group reported that expression of p50 and p52 is not required for formation of RANK-expressing osteoclast progenitors but is essential for RANK-expressing osteoclast precursors to differentiate into osteoclasts in response to RANKL and other osteoclastogenic cytokines [8]. In a rat overiectomized model of estrogen deficiency, administration of NF-κB decoy oligodeoxynucleotides attenuated the increase of TRAP activity, accompanied by a significant increase in calcium concentration in the tibia and femur [9]. A cell-permeable peptide inhibitor of the IκB kinase complex reduced the number of osteoclasts in the joints of collagen-induced arthritic mice [10].

How NF-κB is involved in osteoclastogenesis, however, had not been elucidated until Takatsuna and colleagues demonstrated that DHMEQ suppresses osteoclastogenesis by downregulation of NFATc1 in a culture system of mouse bone marrow-derived monocyte/macrophage precursor cells stimulated with RANKL and M-CSF [12]. The essential role of NFATc1 in osteoclastogenesis was also demonstrated in a recent *in vivo *study using osteoclast-deficient *Fos*^-/- ^mice [34]. In the present study, we found that expression of NFATc1 along the inner surfaces of bone lacunae and eroded bone surface in arthritic joints is suppressed by DHMEQ, suggesting that *in vivo *expression of NFATc1 is significantly regulated by NF-κB in agreement with the *in vitro *studies. RANKL induces NFATc1 expression via three intracellular signaling pathways; an NF-κB pathway, a mitogen-activated protein kinase pathway, and a c-Fos pathway. RANKL also evokes Ca^2+ ^oscillation, which leads to calcineurin-mediated activation of NFATc1 [13]. DHMEQ does not inhibit activation of mitogen-activated protein kinases or inhibit Ca^2+ ^oscillation [12]; the present study therefore also indicates that the NF-κB pathway has priority over other pathways to induce NFATc1 expression.

## Conclusion

*In vivo *administration of the NF-κB inhibitor DHMEQ suppressed differentiation of osteoclasts in collagen-induced mouse arthritis. In addition, DHMEQ exhibited suppressive effects on *in vitro *differentiation and activation of human osteoclasts, suggesting the possible clinical application of this compound.

## Abbreviations

DHMEQ = dehydroxymethylepoxyquinomicin; DMEM = Dulbecco's modified Eagle's medium; ELISA = enzyme-linked immunosorbent assay; FCS = fetal calf serum; FLS = fibroblast-like synovial cells; IL = interleukin; M-CSF = macrophage colony stimulating factor; MMP = matrix metalloprotease; NFAT = nuclear factor of activated T cells; NF = nuclear factor; OPG = osteoprotegerin; PBS = phosphate-buffered saline; RA = rheumatoid arthritis; RANK = receptor activator of NF-κB; RANKL = receptor activator of NF-κB ligand; sRANKL = soluble receptor activator of NF-κB ligand; TNF = tumor necrosis factor; TRAP = tartrate-resistant acid phosphatase

## Competing interests

The authors declare that they have no competing interests.

## Authors' contributions

MH, KA and KO carried out *in vivo *experiments using a mouse model. YK carried out *in vitro *experiments using human cells. KU synthesized a critical chemical. TN and NM participated in the design of the study and helped to draft the manuscript. TK conceived of the study, and participated in its design and drafted the manuscript. All authors read and approved the final manuscript.
